# NAD(P)H: quinone oxidoreductase 1 attenuates oxidative stress and apoptosis by regulating Sirt1 in diabetic nephropathy

**DOI:** 10.1186/s12967-021-03197-3

**Published:** 2022-01-28

**Authors:** Duojun Qiu, Shan Song, Yuhan Wang, Yawei Bian, Ming Wu, Haijiang Wu, Yonghong Shi, Huijun Duan

**Affiliations:** 1 Department of Pathology, Hebei Medical University, No. 361 East Zhongshan Road, Shijiazhuang, 050017 China; 2 Hebei Key Laboratory of Kidney Diseases, Shijiazhuang, China; 3 Digestive Department, Tangshan Workers Hospital, Tangshan, China

**Keywords:** Diabetic nephropathy, NQO1, Sirt1, Oxidative stress, Apoptosis

## Abstract

**Background:**

Diabetic nephropathy (DN) is one of the main complications of diabetes, and oxidative stress plays an important role in its progression. NAD(P)H: quinone oxidoreductase 1 (NQO1) protects cells from oxidative stress and toxic quinone damage. In the present study, we aimed to investigate the protective effects and underlying mechanisms of NQO1 on diabetes-induced renal tubular epithelial cell oxidative stress and apoptosis.

**Methods:**

In vivo, the kidneys of db/db mice, which are a type 2 diabetes model, were infected with adeno-associated virus to induce NQO1 overexpression. In vitro, human renal tubular epithelial cells (HK-2 cells) were transfected with NQO1 pcDNA3.1(+) and cultured in high glucose (HG). Gene and protein expression was assessed by quantitative real-time PCR, western blotting, immunofluorescence analysis, and immunohistochemical staining. Reactive oxygen species (ROS) were examined by MitoSox red and flow cytometry. TUNEL assays were used to measure apoptosis.

**Result:**

In vivo, NQO1 overexpression reduced the urinary albumin/creatinine ratio (UACR) and blood urea nitrogen (BUN) level in db/db mice. Our results revealed that NQO1 overexpression could significantly increase the ratio of NAD+/NADH and silencing information regulator 1 (Sirt1) expression and block tubular oxidative stress and apoptosis in diabetic kidneys. In vitro, NQO1 overexpression reduced the generation of ROS, NADPH oxidase 1 (Nox1) and Nox4, the Bax/Bcl-2 ratio and the expression of Cleaved Caspase-3 and increased NAD+/NADH levels and Sirt1 expression in HK-2 cells under HG conditions. However, these effects were reversed by the Sirt1 inhibitor EX527.

**Conclusions:**

All these data suggest that NQO1 has a protective effect against oxidative stress and apoptosis in DN, which may be mediated by the regulation of Sirt1 through increasing intracellular NAD+/NADH levels. Therefore, NQO1 may be a new therapeutic target for DN.

## Introduction

Diabetic nephropathy (DN) is the main cause of end-stage renal disease (ESRD) in developed countries, and it is a strong predictor of mortality in diabetic patients [1, 2]. The occurrence and development of DN involves many factors, and the mechanism is complex. In the context of hyperglycemia, the overproduction of reactive oxygen species (ROS) and free radicals stimulates the endogenous antioxidant system, reduces glutathione levels and leads to oxidative stress [3, 4]. Excessive intracellular oxidative stress caused by hyperglycemia can occur in mitochondria, which triggers DNA damage and eventually leads to apoptosis in renal cells [5, 6]. In experimental DN, it has been demonstrated that resident renal tubular epithelial cells are lost through apoptosis, and renal tubular epithelial cell apoptosis has been suggested to be related to the progression of proteinuria [7]. Therefore, inhibiting oxidative stress may serve as a potential therapeutic strategy for the treatment of DN.

Cellular nicotinamide adenine dinucleotide (NAD) is a metabolic cofactor that exists in oxidized (NAD+) or reduced (NADH) forms. NAD+ is a cofactor of a variety of enzymes, including sirtuins (Sirts), cyclic ADP (CADP)-ribose synthetase and poly (ADP-ribose) transferase (PARP) [8, 9], and regulating NAD+ levels may play a therapeutic role by affecting NAD+ -dependent enzymes. Silencing information regulator 1 (Sirt1) is a Sirt family member that plays a critical role in many biological processes, including resisting oxidative stress and apoptosis and inhibiting inflammation [10]. The Sirt1 activator resveratrol ameliorates renal cell apoptosis in streptozotocin-induced diabetic rats and HK-2 cells under hyperglycemic conditions [11]. Similarly, resveratrol increased the deacetylase activity of Sirt1 and improved renal tubular oxidative stress damage induced by hyperglycemia [12]. Therefore, modulating the activity and expression of Sirt1 plays a key role in preventing the progression of diabetic kidney injury.

NAD(P)H: quinone oxidoreductase 1 (NQO1) is a cytoplasmic antioxidant flavin that increases the levels of intracellular NAD+ by using NADH as an electron donor to catalyze the reduction of quinone to hydroquinone [13, 14]. Notably, intracellular free NAD+ levels are decreased under various pathological conditions, including hypertension, arterial resting contraction, aging and diabetes [15, 16]. Moreover, recent studies have shown that NQO1 is activated by β-lapachone and has many beneficial effects, such as improving obesity or hypertension, preventing arterial restenosis or health decline in aging mice and blocking salt-induced renal injury [17–22]. Pharmacological activation of NQO1 alleviates cisplatin-induced renal oxidative stress and inflammation by increasing intracellular NAD+ levels [23]. However, the role of NQO1 in oxidative stress and apoptosis in DN has not been elucidated.

In this study, we observed the expression of NQO1 in patients with DN and used type 2 diabetic mice (db/db mice) to investigate the effects of NQO1 overexpression on renal function, oxidative stress and apoptosis in diabetic kidneys. Furthermore, we explored the molecular mechanism by which NQO1 regulates oxidative stress and apoptosis in HK-2 cells cultured with high glucose.

## Materials and methods

### Antibodies and other reagents

Antibodies against NQO1 (11451-1-AP), Nox1 (17772-1-AP), Bax (50599-2-IG), Nox4 (14347-1-AP), Sirt1 (13161-1-AP) and β‐actin (20536-1-AP) were purchased from Proteintech (Chicago, IL, USA). 8-Hydroxy-2'-deoxyguanosine (8-OHdG) (ab48508) and Bcl-2 (ab32124) were purchased from Abcam (Cambridge, UK). The Cleaved Caspase-3 (9664) antibody was obtained from Cell Signaling Technology (Danvers, MA). The culture medium, fetal bovine serum (FBS) and Dulbecco's modified Eagle's medium (DMEM)-F12 were obtained from Gibco Company (Gaithersburg, MD). The plasmids for NQO1 pcDNA3.1(+) and control pcDNA3.1(+) were obtained from Gene Pharma (Shanghai, China). The transfection reagent FuGENE-HD was obtained from Promega (Madison, Wisconsin, USA). TRIzol was purchased from Invitrogen (Carlsbad, CA). Takara (Shiga, Japan) provided SYBR Premix Ex Taq II. EX527 was purchased from Selleckchem (Shanghai, China). A TUNEL FITC apoptosis detection kit was purchased from Vazyme Biotech Corporation (Nanjing, China). Adeno-associated virus serotype 9 (AAV9) was obtained from HanBio Technology (HH20191024HBYXL-AAV01, Shanghai, China).

### Animals and treatments

Eight-week-old male C57BL/KsJ db/db mice and littermate control db/m mice (n = 8) were obtained from Nanjing University (Nanjing, China). The mice were kept in a temperature-controlled room at 22 ± 2 °C with a light/dark cycle of 12 h, and standard food and water were freely available. All experiments were approved by the Institutional Animal Care and Use Committee of Hebei Medical University. The db/db mice were randomly divided into three groups: db/db group (n = 8), db/db + AAV-Control group (AAV empty vector-treated db/db mice, n = 8), db/db + AAV-NQO1 group (AAV-NQO1-treated db/db mice, n = 8). To assess the effect of NQO1 overexpression on diabetic kidneys, db/db mice were treated with an NQO1 overexpression AAV9 vector. A total of 50 μL of 1 × 10^11^ infective units of AAV-Control or AAV-NQO1 was injected into three sites in the renal cortex of each kidney of db/db mice and after 12 weeks, the mice were sacrificed at the age of 20 weeks [24–26]. Subsequently, 24-h urine, blood samples and renal cortex tissues were collected for analysis.

### Human renal biopsies

Human renal biopsies were collected from the Second Hospital of Hebei Medical University. The research protocol for human tissues is consistent with the principles of the Helsinki Declaration and was approved by the Clinical Research Ethics Committee of Hebei Medical University. Informed consent was obtained from patients according to approved guidelines. In this study, 15 kidney biopsies from patients were collected, including 10 samples from patients with type 2 diabetes mellitus with nephropathy and 5 normal renal tissue samples from distal kidney resection because of the presence of localized tumors. Renal biopsies were performed in the DN group to rule out the possibility of other renal diseases.

### Cell culture and transfection

HK-2 cells (American Type Culture Collection, Manassas, VA, USA) were cultured in DMEM-F12 (3:1) supplemented with 10% FBS, 100 U/mL penicillin, and 100 U/mL streptomycin in a 95% air and 5% CO_2_ atmosphere at 37 °C. After the cells reached 40–50% confluence, they were pre-treated with serum-free medium for 12 h and then transfected with NQO1 pcDNA3.1(+) or control pcDNA3.1(+) plasmid with FuGENE-HD transfection reagent in HG medium for 48 h. HK-2 cells were stimulated with normal glucose (NG, 5.6 mmol/L), high glucose (HG, 30 mmol/L), NG plus mannitol (M, 24.4 mmol/L), HG plus control pcDNA3.1(+) (HG + C), HG plus NQO1 pcDNA3.1(+) (HG + NQO1 O/E), and HG plus NQO1 pcDNA3.1(+) and EX527 (HG + NQO1 O/E + EX527; 1 μM) for 48 h.

### Western blot analysis

Total proteins (30–50 µg) were isolated from renal tissue and HK-2 cells with RIPA buffer (Solarbio, Beijing, China) containing a protease-phosphatase inhibitor mixture. The proteins were collected by centrifugation at 12,000 r at 4 °C for 20 min, and the concentration was determined by a BCA protein analysis kit (Solarbio, Beijing, China). For immunoblotting, equal amounts of protein were resolved by SDS-PAGE, transferred onto polyvinylidene fluoride (PVDF) membranes (Burlington Millipore, MA, USA) and sealed with 5% skim milk at 37 °C for 1 h. Then, the blots were incubated at 4 °C overnight with primary antibodies against NQO1, Bax, Bcl-2, Nox1, Nox4, Cleaved Caspase-3, Sirt1 and β-actin. Incubation with secondary antibodies was performed at 37 °C for 1 h. After the blots were washed with TBS with Tween-20 (TBST), the bands were detected by an ECL reagent and scanned using a GE-Amersham Imager 600 (General Electric Company, USA). Band densitometry was assessed by National Institutes of Health (NIH) ImageJ 1.50 software.

### RNA extraction and quantitative RT-qPCR analysis

Using TRIzol reagent (Invitrogen), total RNA was extracted from kidney tissues or HK-2 cells, and cDNA was prepared using a reverse transcription kit (Promega, Madison, WI, USA) according to the instructions. Real-time PCR was performed using SYBR Premix Ex Taq II on an Agilent Mx3000P qPCR System (Agilent, CA), and GAPDH served as an internal control [27, 28]. The primers used were as follows: NQO1 (human), F: ATGTATGACAAAGGACCCTTCC and R: TCCCTTGCAGAGAGTACATGG; Sirt1 (human), F: CAGTGTCATGGTTCCTTTGC and R: CACCGAGGAACTACCTGAT; GAPDH (human), F: CTGACTTCAACAGCGACACC and R: TGCTGTAGCCAAATTCGTTGT; NQO1 (mouse), F: AGGATGGGAGGTACTCGAATC and R: TGCTAGAGATGACTCGGAAGG; Sirt1 (mouse), F: TCAGAGTTGCCACCAACAC and R: TACTGGAACCAACAGCCTTA; and GAPDH (mouse), F: CGGAGTCAACGGATTTGGTCGTAT and R: AGCCTTCTCCATGGTGGTGAAGAC. The relative expression of each target gene was calculated by the 2^−ΔΔCT^ method.

### Immunohistochemistry

Kidney tissues were fixed in 4% paraformaldehyde and embedded in paraffin. The tissue sections (4 μm) were deparaffinized with xylene and rehydrated with gradient alcohol. Internal peroxidase activity was inactivated using 3% hydrogen peroxide in 100% methanol for 20 min at room temperature after antigen retrieval for 5 min at 121 °C using 10 mM citrate buffer (pH 6.0). Next, the sections were incubated with 10% normal goat serum for 30 min at room temperature to block nonspecific antibody binding and were incubated with primary antibodies against NQO1, Bax, Bcl-2, cleaved caspase-3, Nox1, Nox4, 8-OHdG and Sirt1 overnight at 4 °C. After being washed with phosphate-buffered saline (PBS), the slices were incubated with biotinylated secondary antibodies and horseradish peroxidase-conjugated streptavidin at 37 °C for 30 min. Labeling was visualized with diaminobenzidine (DAB) at room temperature for 1–2 min to produce a brown color, and the sections were counterstained with hematoxylin. Finally, the images were captured with an Olympus microscope (Olympus, BX71, Tokyo, Japan). The average integrated optical density was quantified by Image-Pro Plus 6.0 (Media Cybernetics) software to indicate protein expression.

### Periodic acid-Schiff and Masson trichrome staining

Renal tissue sections (4 μm) were subjected to periodic acid-Schiff (PAS) staining to identify glycogen deposition and Masson trichrome staining to identify collagen deposition. Semiquantitative indicators were used to assess the extent of glomerular mesangial dilatation and sclerosis [29]. In short, 10 nonoverlapping regions in each kidney section were randomly selected for examination. The grading of each glomerulus in a single section ranged from 0 to 4, with 0 representing no lesion and 1, 2, 3 and 4 representing the expansion or hardening of mesangial matrix, involving 25%, 25% to 50%, 50% to 75% or > 75% of glomerular tuft area, respectively. Collagen-positive areas in the kidney were measured by NIH ImageJ 1.50 software.

### Immunofluorescence analysis

HK-2 cells were grown on slides in a six-well chamber, fixed with 4% paraformaldehyde for 40 min at 4 °C and stabilized with 0.15% Triton X-100 for 10 min at room temperature. After 30 min goat serum blocking at 37 °C, HK-2 cells were incubated with antibodies against NQO1 (1:150), Sirt1 (1:150), Bcl-2 (1:200), Cleaved Caspase-3 (1:200), Nox1 (1:100), Nox4 (1:200) and Bax (1:150) overnight at 4 °C. The cells were incubated with FITC-labeled secondary antibodies (1:150) for 1 h at 37 °C. Then, the slides were washed with PBS three times, and cell nuclei were stained with DAPI for 10 min. Finally, the slides were observed under a confocal microscope (Leica, Germany). ImageJ 1.50 software from the NIH was used to evaluate the collected images.

### ROS analysis

Mitochondrial ROS were measured by MitoSox red (Invitrogen). Cells were cultured in a six-well chamber for 48 h and subsequently incubated with MitoSox red at a final concentration of 5 μM in the dark for 30 min at 37 °C. Then, the cells were washed three times with Hank’s balanced salt solution (HBSS), and images were acquired using a confocal microscope (Leica, Germany). The fluorescence intensity of mitochondrial ROS was quantified using Image-Pro Plus 6.0 software (Media Cybernetics). For flow cytometry, DCFH-DA (Invitrogen) was used to assess intracellular ROS levels. HK‐2 cells were treated with 10 μM DCFH-DA for 30 min in a dark incubator at 37 °C. After being washed with PBS, the ROS levels were measured by a flow cytometer (FACS Aria, BD Biosciences, CA, USA).

### TUNEL assay

Terminal deoxynucleotidyl transferase-mediated dUTP nick end labeling (TUNEL) in renal tissues and cultured HK‐2 cells was measured by an apoptosis detection kit (Vazyme, China). Fluorescence images were obtained by a confocal microscope (Leica, Germany). TUNEL-positive apoptotic cells were counted in six different fields (×400) for each sample and then averaged.

### Sirt1 activity assay

The activity of Sirt1 in renal tissue and HK-2 cells was determined using a fluorometric Sirt1 assay kit (Sigma–Aldrich) [30]. In brief, samples were incubated with 10 μL of Sirt1 substrate solution in the presence or absence of NAD+. Samples were incubated at 37 °C for 1 h. Next, 5 μL of developing buffer was added to each well, and samples were incubated at 37 °C for 10 min. The fluorescence intensities were measured using a microplate fluorometer (excitation wavelength = 360 nm, emission wavelength = 450 nm).

### Determination of the NAD+/NADH ratio

NAD+/NADH was measured using the NAD+/NADH assay kit (Beyotime, China) according to the manufacturer’s instructions. In brief, the tissues or cells were homogenized in 200 μL of NAD+/NADH extraction buffer. After neutralization, the total amount of intracellular NAD+ and NADH was measured. After being heated at 60 °C for 30 min, intracellular NADH levels were measured, and the optical density was determined at 450 nm.

### Statistical analysis

The data are expressed as the mean ± SD. Student’s t test was used to analyze two groups, and one-way ANOVA was used to analyze no less than three groups. A value of *P* < 0.05 indicated that the results were statistically significant.

## Results

### NQO1 overexpression attenuated renal interstitial fibrosis and renal function in db/db mice

**Fig. 1:**
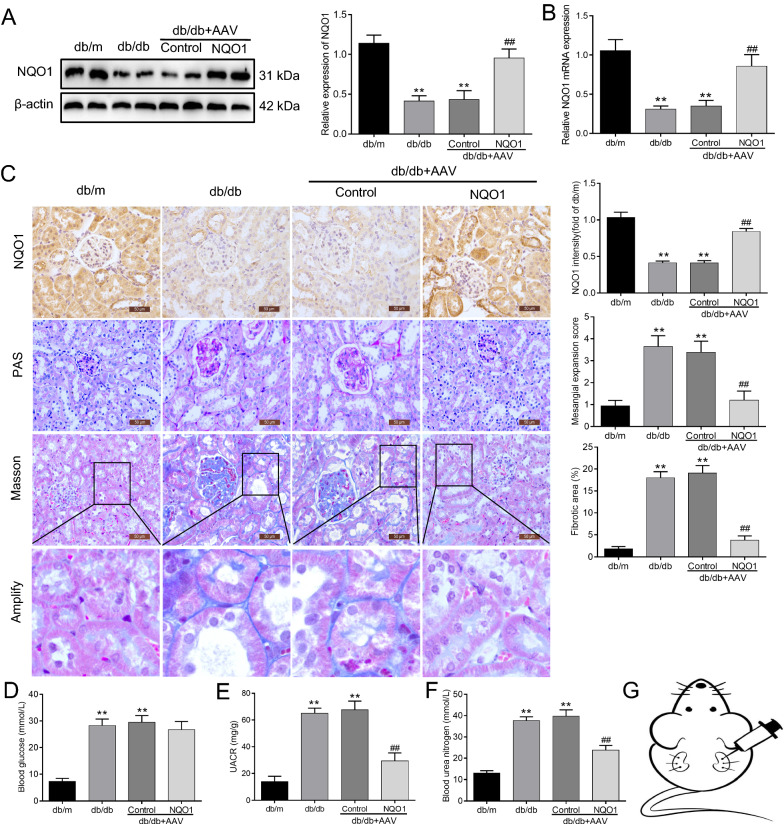
NQO1 overexpression attenuated renal interstitial fibrosis and renal function in db/db mice. **A–C** The effect of AAV-NQO1 on the expression of NQO1 in db/db mice was measured by western blotting (n = 6), RT-qPCR (n = 6) and immunohistochemical staining (scale bar, 50 μm, n = 9). **C** Kidney sections were stained with periodic acid‐Schiff (PAS) and Masson trichrome (Masson) (scale bar, 50 μm, n = 9), and mesangial expansion and fibrotic areas were measured. Changes in **D** blood glucose concentrations (n = 8), **E** UACR and **F** BUN were measured in the different groups (n = 6). **G** The kidneys of db/db mice in the db/db + AAV-Control group (n = 8) and db/db + AAV-NQO1 group (n = 8) were injected with 50 μL of 1 × 10^11^ infective units of AAV-Control or AAV-NQO1 at three sites. NQO1: NAD(P)H: quinone oxidoreductase 1, AAV: adeno-associated virus, RT-qPCR: reverse transcription-quantitative polymerase chain reaction, UACR: urine albumin/creatinine ratio BUN: blood urea nitrogen. Values are expressed as the mean ± SD. **P < 0.01 versus the db/m group; ^##^*P* < 0.01 versus the db/db + AAV-Control group

To determine the effect of NQO1 overexpression on diabetic renal injury, an AAV9 vector was injected into the kidneys of db/db mice to regulate the expression of NQO1 (Fig. 1G). As shown in Fig. 1A and B, compared with that in db/m mice, the protein and mRNA expression of NQO1 in the kidneys of db/db mice was notably decreased, while NQO1 protein and mRNA expression in the kidneys of db/db mice treated with AAV-NQO1 were increased by 2.31 and 2.46 times versus AAV-Control-treated db/db mice, respectively (*P* < 0.01). Immunohistochemical staining showed that NQO1 was mainly expressed in the cytoplasm of renal tubular epithelial cells; compared with that in AAV-Control-treated db/db mice, NQO1 expression, as indicated by brown granules, was markedly enhanced in the kidneys of db/db mice treated with AAV-NQO1 (Fig. 1C). PAS and Masson’s trichrome staining were used to evaluate mesangial expansion and renal fibrosis. The results showed that NQO1 overexpression markedly ameliorated mesangial expansion and renal interstitial fibrosis in db/db mice (Fig. 1C). The blood glucose levels of db/db mice were significantly enhanced compared with those of db/m mice, but there was no difference in blood glucose levels between the db/db + AAV-Control and db/db + AAV-NQO1 groups (Fig. 1D). The urinary albumin/creatinine ratio (UACR) and blood urea nitrogen (BUN) were significantly elevated in db/db mice compared with db/m mice. However, db/db mice treated with AAV-NQO1 showed improvements in renal function. Compared with the db/db + AAV-Control group, the db/db + AAV-NQO1 group had obvious decreases in the UACR and BUN (Fig. 1E, F). These results demonstrated that NQO1 overexpression could ameliorate renal interstitial fibrosis and renal function in db/db mice.

### NQO1 overexpression reduced renal Nox1 and Nox4 expression and renal cell apoptosis in db/db mice

**Fig. 2:**
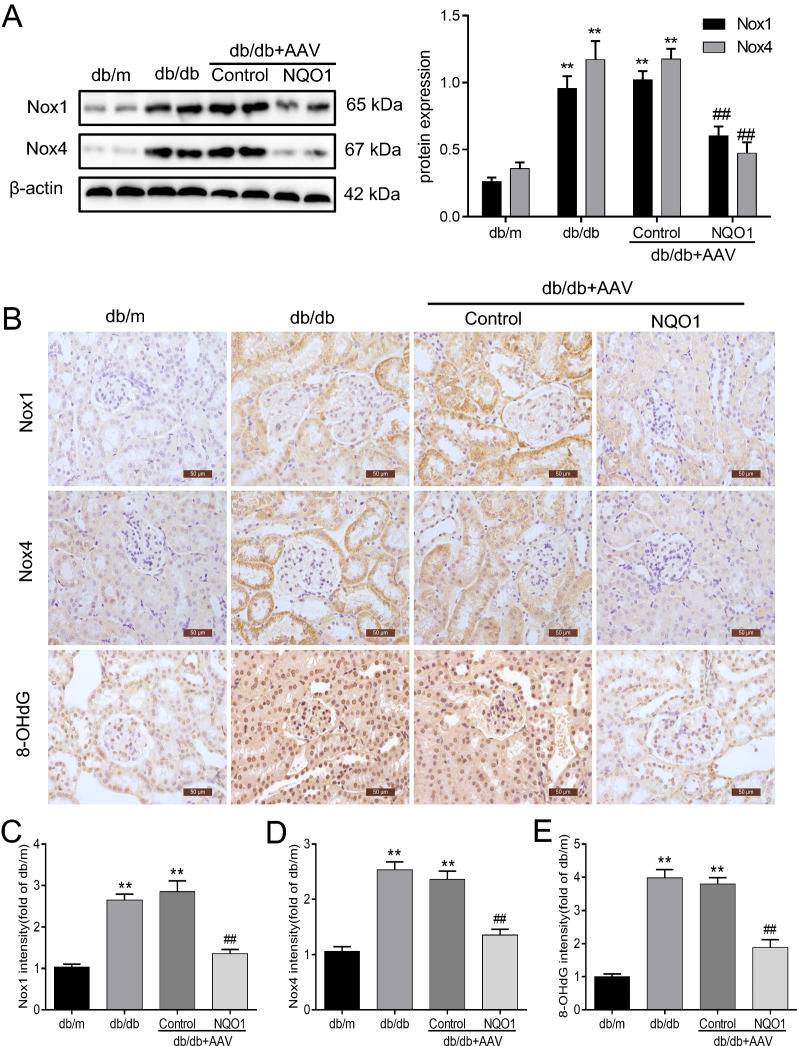
NQO1 overexpression suppresses oxidative stress in diabetic kidneys. **A** The protein expression of Nox1 and Nox4 was evaluated by western blotting (n = 6). **B–E** Immunohistochemical staining analysis of Nox1, Nox4 and 8-OHdG expression in the renal tissues of mice (scale bar, 50 μm, n = 9). 8-OHdG: 8-Hydroxy-2'-deoxyguanosine. Values are expressed as the mean ± SD. **P < 0.01 versus the db/m group; ^##^*P* < 0.01 versus the db/db + AAV-Control group

As shown in Fig. 2A, the protein levels of Nox1 and Nox4 in db/db mice were evidently upregulated compared with those in db/m mice, and renal Nox1 and Nox4 protein levels in db/db mice with AAV-NQO1 treatment were reduced by 41.23% and 58.84%, respectively, compared with those in mice with AAV-Control treatment (*P* < 0.01). Furthermore, immunohistochemical staining of Nox1 and Nox4 was performed in renal tissue sections. The protein expression of Nox1 and Nox4 in the renal tubules of db/db mice was significantly higher than that of db/m mice. Importantly, Nox1 and Nox4 expression in diabetic kidneys was inhibited by NQO1 overexpression (Fig. 2B–D). Moreover, the level of 8-OHdG, a marker of oxidative stress, was measured in renal tissues. The results showed that nuclear 8‐OHdG expression in the kidneys of db/db mice was markedly increased, and this level decreased after AAV-NQO1 treatment (Fig. 2B, E).

**Fig. 3:**
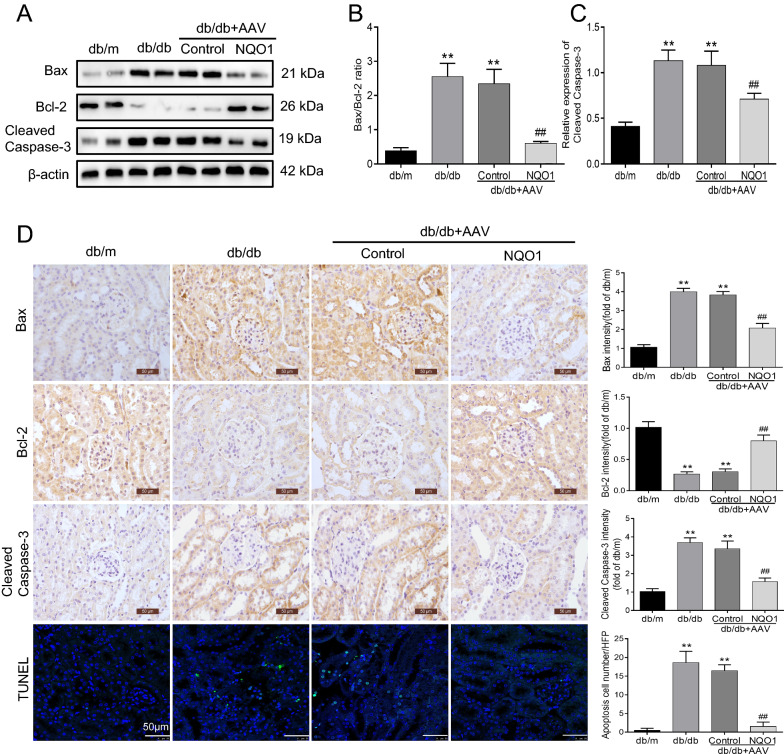
NQO1 overexpression reduced renal cell apoptosis. **A–C** The expression of Bax, Bcl-2, and Cleaved Caspase-3 was measured by western blotting (n = 6). **D** Immunohistochemical staining of Bax, Bcl-2, and Cleaved Caspase-3 in kidney sections (scale bar, 50 μm, n = 9). **D** Apoptosis was determined using the TUNEL assay, and **D** the mean apoptotic cells per field was determined (magnification, × 400, n = 9). Values are expressed as the mean ± SD. **P < 0.01 versus the db/m group; ^##^*P* < 0.01 versus the db/db + AAV-Control group

Furthermore, we examined the effect of NQO1 overexpression on renal cell apoptosis in db/db mice. The Bax/Bcl-2 ratio and protein level of Cleaved Caspase-3 were markedly upregulated in the renal tissues of db/db mice compared with those of db/m mice. NQO1 overexpression notably inhibited diabetes-induced Cleaved Caspase-3 expression and reversed the ratio of Bax to Bcl-2 in kidneys. Statistical analysis revealed that the Bax/Bcl-2 ratio was decreased by 74.31% in db/db mice with AAV-NQO1 treatment versus AAV-Control treatment (*P* < 0.01). Similarly, Cleaved Caspase-3 expression was reduced by 30.76% in db/db mice treated with AAV-NQO1 versus AAV-Control (*P* < 0.01) (Fig. 3A–C). Immunohistochemistry also confirmed that AAV-NQO1 treatment upregulated the expression of Bcl-2 and downregulated the expression of Bax and Cleaved Caspase-3, as indicated by brown granules in the renal tubular cells of db/db mice (Fig. 3D). In addition, TUNEL staining revealed obvious renal tubular cell apoptosis of renal tubular cells in db/db mice. However, after AAV-NQO1 treatment in db/db mice, a few renal cells were TUNEL-positive (Fig. 3D). Overall, these results demonstrated that NQO1 overexpression reduced Nox1 and Nox4 expression and renal cell apoptosis in db/db mice.

### NQO1 overexpression upregulated renal Sirt1 expression and activity in db/db mice

**Fig. 4:**
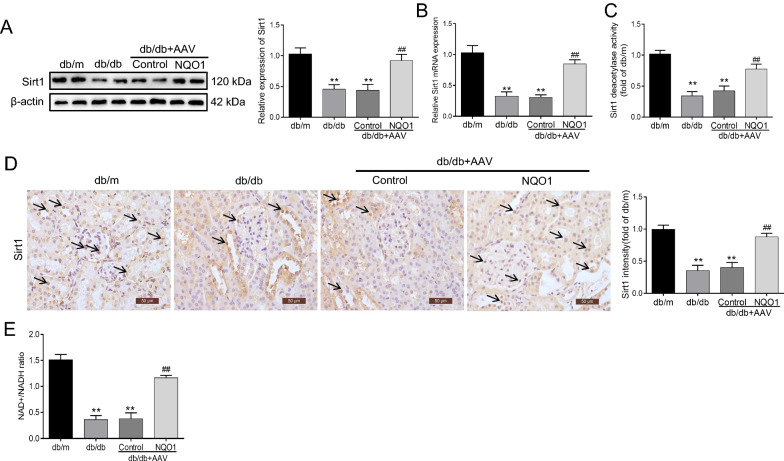
NQO1 overexpression upregulated renal Sirt1 expression and activity in db/db mice. **A**, **B** The expression of Sirt1 was measured by western blotting (n = 6) and RT-qPCR (n = 6). (**C**) Sirt1 activity in renal tissues (n = 6). **D** Sirt1 was measured by immunohistochemical staining in db/db mice (scale bar, 50 μm, n = 9). **E** The intracellular NAD+/NADH ratio was measured in db/db mice (n = 6). Sirt1: Silencing information regulator 1. Values are expressed as the mean ± SD. **P < 0.01 versus the db/m group; ^##^*P* < 0.01 versus the db/db + AAV-Control group

We further examined the effect of NQO1 overexpression on Sirt1 expression and activity in the kidneys of db/db mice. As shown in Fig. 4A–C, the expression and activity of Sirt1 were notably decreased in diabetic kidney tissues, whereas these effects were clearly reversed by AAV-NQO1 treatment. Statistical analysis confirmed that the levels of Sirt1 protein, mRNA and activity were increased by 109.52%, 178.10% and 82.75%, respectively, in db/db mice treated with AAV-NQO1 versus AAV-Control (*P* < 0.01). In addition, immunohistochemical staining revealed that Sirt1 was expressed in the nuclei of glomeruli and renal tubules, and the expression of Sirt1 in the kidneys of db/db mice was lower than that in the kidneys of db/m mice; AAV-NQO1 treatment significantly enhanced renal Sirt1 expression in db/db mice (Fig. 4D). Next, we evaluated the effect of NQO1 overexpression on the proportion of cellular NAD+/NADH in the kidneys of db/db mice. Figure 4E shows that the ratios of cellular NAD+/NADH were reduced in db/db mice compared to db/m mice; db/db mice treated with AAV-NQO1 had upregulated cellular NAD+/NADH ratios in the kidneys. The cellular NAD+/NADH ratio in the kidneys was increased by 2.12 times in db/db mice with AAV-NQO1 treatment versus AAV-Control treatment (*P* < 0.01). Taken together, these results showed that NQO1 overexpression increased renal Sirt1 expression and activity in db/db mice by regulating NAD+/NADH levels.

### NQO1 overexpression ameliorated HG-induced oxidative stress in HK‐2 cells

**Fig. 5:**
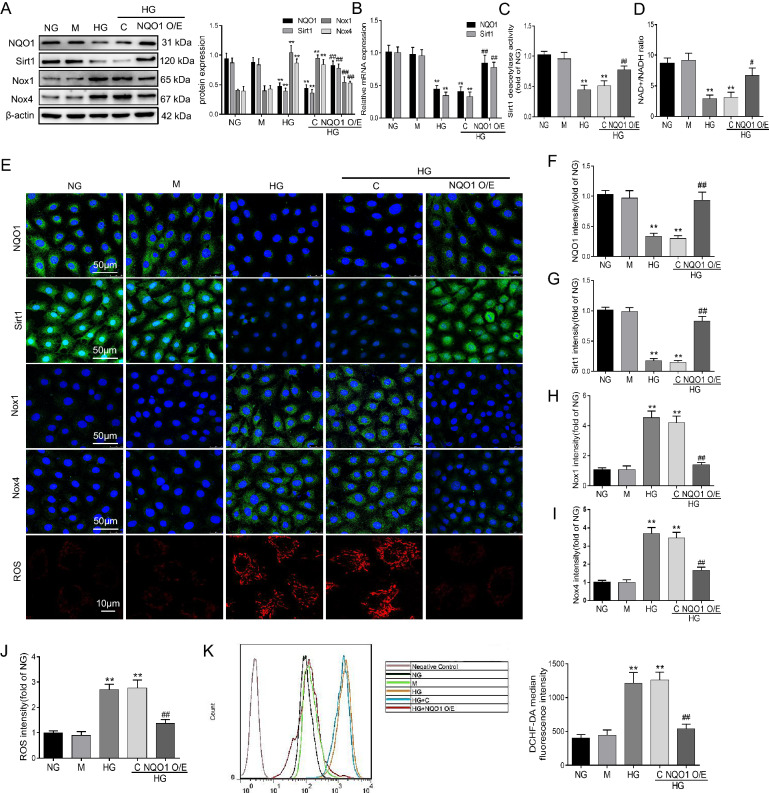
Role of NQO1 overexpression in oxidative stress in HG-cultured HK-2 cells. **A** The protein levels of NQO1, Sirt1, Nox1 and Nox4 were analyzed by western blotting (n = 3). **B** The mRNA levels of NQO1 and Sirt1 were measured by RT-qPCR (n = 3). **C** Sirt1 activity in HK-2 cells (n = 6). **D** The intracellular NAD+/NADH ratio was measured in HK-2 cells (n = 6). **E–I** The expression levels of NQO1, Sirt1, Nox1 and Nox4 were measured by immunofluorescence (scale bar, 50 μm, n = 9). **E** Mitochondrial ROS was assessed by the fluorescence probe MitoSOX Red (scale bar, 10 μm, n = 6). **J** Quantitative analysis of mitochondrial ROS. **K** Intracellular ROS were measured by flow cytometry and quantitative analysis (n = 6). NG: 5.6 mM d‐glucose, M: NG + mannitol (24.4 mM), HG: 30 mM d‐glucose, HG + C: HG + control pcDNA3.1(+), HG + NQO1 O/E: HG + NQO1 pcDNA3.1(+). Values are expressed as the mean ± SD. **P < 0.01 versus NG group; ^#^*P* < 0.05, ^##^*P* < 0.01 versus HG + C group

Animal experiments confirmed that NQO1 overexpression reduced renal tubular cell apoptosis and oxidative stress. Therefore, to further elucidate the function of NQO1 in renal tubular cells in diabetes mellitus, we overexpressed NQO1 in HK-2 cells treated with HG using the pcDNA3.1(+) plasmid targeting NQO1. As shown in Fig. 5A, B, the western blot and RT-qPCR results showed that NQO1 and Sirt1 expression was decreased in HK-2 cells treated with HG for 48 h compared to those treated with NG, and the HG‐induced reduction in NQO1 and Sirt1 expression was reversed by NQO1 pcDNA3.1(+). Statistical analysis revealed that NQO1 protein and mRNA increased by 1.83 and 2.07 times, respectively, and Sirt1 protein and mRNA increased by 2.04 and 2.36 times, respectively, in HG-induced HK-2 cells transfected with NQO1 pcDNA3.1(+) compared with those transfected with control pcDNA3.1(+) (*P* < 0.01). Furthermore, as shown in Fig. 5C, NQO1 overexpression obviously increased Sirt1 activity in HK-2 cells under HG conditions. We then measured the cellular NAD+/NADH ratio in HK-2 cells under HG conditions. Compared with that in the NG group, the cellular NAD+/NADH ratio decreased in the HG group, and NQO1 overexpression reversed the cellular NAD+/NADH ratio in HG-induced HK-2 cells. In detail, the NAD+/NADH ratio was enhanced 2.26 times in HG-cultured HK-2 cells transfected with NQO1 pcDNA3.1(+) (*P* < 0.05) (Fig. 5D). Similar to the western blot results, the immunofluorescence results also confirmed that NQO1 pcDNA3.1(+) transfection upregulated NQO1 and Sirt1 expression, as indicated by the green granules in HG-cultured HK-2 cells (Fig. 5E–G).

To explore the role of NQO1 in HG-induced oxidative stress in HK-2 cells, the protein expression of Nox1 and Nox4 was measured by western blotting after the cells were incubated with HG for 48 h. The protein levels of Nox1 and Nox4 in HK-2 cells were markedly upregulated under HG conditions compared to those in the NG group, while the increases in Nox1 and Nox4 were evidently suppressed by NQO1 pcDNA3.1(+) plasmid transfection. In detail, Nox1 and Nox4 were decreased by 42.17% and 36.79%, respectively, in HG-cultured HK-2 cells transfected with NQO1 pcDNA3.1(+) compared to control pcDNA3.1(+) (*P* < 0.01) (Fig. 5A). In addition, the immunofluorescence results were similar to the western blot results, in which NQO1 pcDNA3.1(+) transfection decreased Nox1 and Nox4 protein expression in HG-cultured HK-2 cells (Fig. 5E, H, I). Next, after transfection with NQO1 pcDNA3.1(+), the level of ROS in mitochondria induced by HG decreased significantly, as indicated by red fluorescence in HG-exposed HK-2 cells (Fig. 5E, J). Moreover, we found that ROS levels increased after HG treatment and were significantly decreased by NQO1 overexpression in HK-2 cells, as demonstrated by the DCFH-DA assay (Fig. 5K).

### NQO1 overexpression abolished HG-induced apoptosis in HK-2 cells

**Fig. 6:**
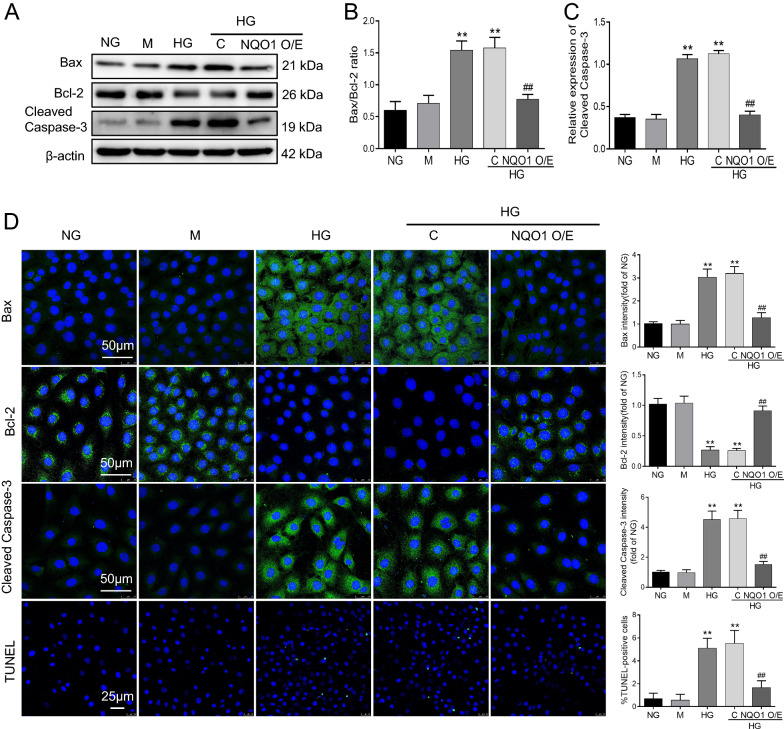
Effect of NQO1 overexpression on HG-induced apoptosis in HK-2 cells. **A–C** The protein levels of Bax, Bcl-2 and Cleaved Caspase-3 were analyzed by western blotting (n = 3). **D** The expression levels of Bax, Bcl-2 and Cleaved Caspase-3 were measured by immunofluorescence (scale bar, 50 μm, n = 6). **D** Apoptosis in HK-2 cells was determined by TUNEL assays and the percentage of TUNEL-positive cells per field (× 400) (Scale bar, 50 μm, n = 6). NG: 5.6 mM d‐glucose, M: NG + mannitol (24.4 mM), HG: 30 mM d‐glucose, HG + C: HG + control pcDNA3.1(+), HG + NQO1 O/E: HG + NQO1 pcDNA3.1(+). Values are expressed as the mean ± SD. **P < 0.01 versus NG group; ##*P* < 0.01 versus HG + C group

As shown in Fig. 6A–C, the results revealed that the Bax/Bcl-2 ratio and Cleaved Caspase-3 expression were markedly increased in HG-exposed HK-2 cells, and this effect was significantly attenuated by NQO1 pcDNA3.1(+) plasmid transfection. Statistical analysis revealed a 51.2% decrease in the Bax/Bcl-2 ratio and a 64.4% decrease in Cleaved Caspase-3 in HG-induced HK-2 cells transfected with the NQO1 pcDNA3.1(+) plasmid versus control pcDNA3.1(+) plasmid (*P* < 0.01). Moreover, we evaluated the protein expression of Bax, Bcl-2, and Cleaved Caspase-3 by immunofluorescence in HK-2 cells, and the immunofluorescence results were similar to the western blot results. NQO1 pcDNA3.1(+) plasmid transfection significantly increased Bcl-2 expression and decreased Bax and Cleaved Caspase-3 expression, as indicated by green fluorescence in HG-exposed HK-2 cells (Fig. 6D). Similarly, TUNEL staining revealed that cellular apoptosis in HG-exposed HK-2 cells was markedly enhanced compared with that in nontreated cells, which could be blocked by NQO1 overexpression (Fig. 6D).

### The Sirt1 inhibitor EX527 ameliorated the inhibitory effect of NQO1 overexpression on oxidative stress in HG-cultured HK-2 cells

**Fig. 7:**
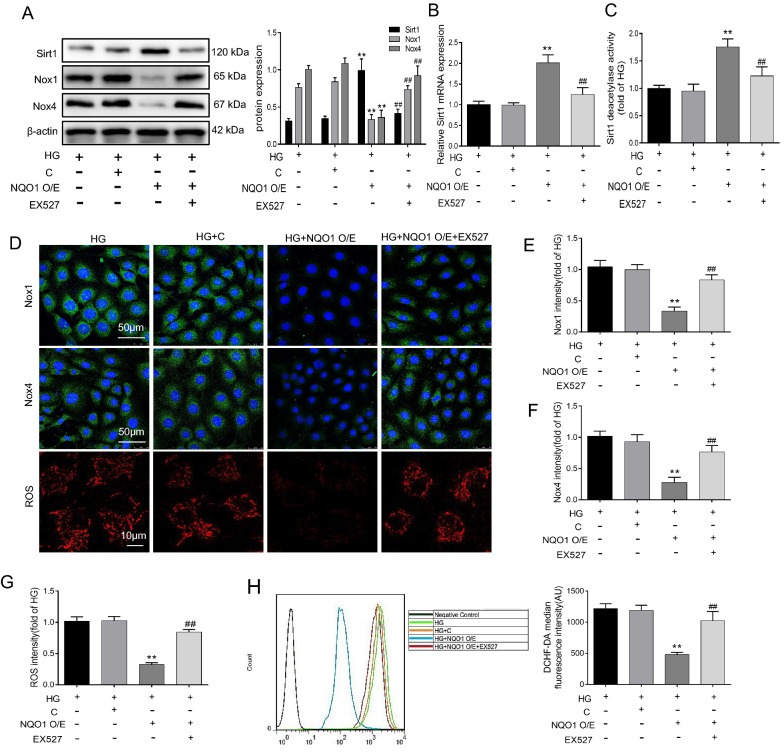
Effect of EX527 on oxidative stress in NQO1 pcDNA-treated HK-2 cells under HG conditions. **A** The protein levels of Sirt1, Nox1 and Nox4 were analyzed by western blotting (n = 3). **B** The mRNA level of Sirt1 was measured by RT-qPCR (n = 3). (**C**) Sirt1 activity in HK-2 cells (n = 6). **D–F** The expression levels of Nox1 and Nox4 were measured by immunofluorescence (scale bar, 50 μm, n = 6). **D** Mitochondrial ROS was assessed by the fluorescence probe MitoSOX red (Scale bar, 10 μm, n = 6). **G** The quantitative analysis of mitochondrial ROS. (**H**) Intracellular ROS was detected by flow cytometry and quantitative analysis was performed (n = 6). HG: 30 mM d‐glucose, HG + C: HG + control pcDNA3.1(+), HG + NQO1 O/E: HG + NQO1 pcDNA3.1(+), HG + NQO1 O/E + EX527: HG + NQO1 pcDNA3.1(+) + EX527 (1 μM). Values are expressed as the mean ± SD. **P < 0.01 versus HG + C group; ^##^*P* < 0.01 versus HG + NQO1 O/E group

To further verify whether Sirt1 regulates oxidative stress and apoptosis via NQO1, we used the Sirt1 inhibitor EX527 in HG-induced HK-2 cells transfected with NQO1 pcDNA3.1(+). The results suggested that NQO1 overexpression efficiently enhanced Sirt1 expression and activity in HG-induced HK-2 cells, but these changes could be abolished by EX527 (Fig. 7A–C). In detail, the levels of Sirt1 protein, mRNA and activity were reduced by 51.86%, 65.25% and 43.21%, respectively, in EX527-treated HG-cultured HK-2 cells transfected with NQO1 pcDNA3.1(+) compared with those in NQO1 pcDNA3.1(+)-transfected HG-cultured HK-2 cells (*P* < 0.01). Additionally, Nox1 and Nox4 expression were decreased by the NQO1 pcDNA3.1(+) plasmid in HG-exposed HK-2 cells, and this effect was blocked by EX527 (Fig. 7A). After EX527 treatment, Nox1 and Nox4 protein expression was enhanced by 2.21- and 2.56-fold, respectively, in HG-exposed HK-2 cells transfected with the NQO1 pcDNA3.1(+) plasmid (*P* < 0.01). Furthermore, immunofluorescence analysis confirmed the western blot results, and EX527 treatment increased Nox1 and Nox4 expression, as indicated by green granules, in HG-exposed HK-2 cells transfected with the NQO1 pcDNA3.1(+) plasmid (Fig. 7D–F). MitoSOX red staining and flow cytometry revealed that NQO1 overexpression suppressed HG-induced ROS generation and that these results could be inhibited by EX527 (Fig. 7D, G, H). Taken together, these results indicate that NQO1 overexpression alleviates HG-induced oxidative stress in HK‐2 cells and is related to the activation of Sirt1.

### EX527 abrogated the antiapoptotic effect of NQO1 overexpression on HG-induced HK-2 cells

**Fig. 8:**
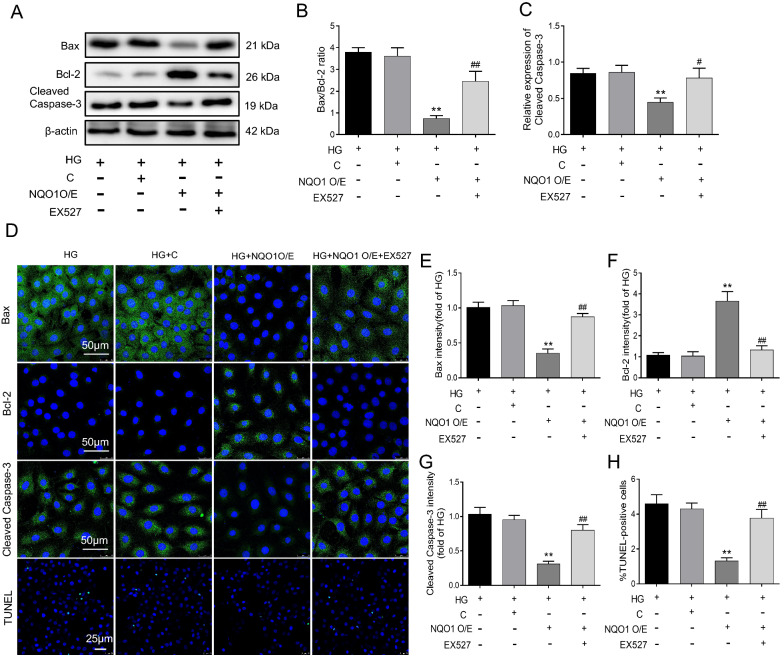
Effect of EX527 on apoptosis in NQO1 pcDNA-treated HK-2 cells under HG conditions. **A–C** The protein levels of Bax, Bcl-2 and Cleaved Caspase-3 were analyzed by western blotting (n = 3). **D–G** The protein expression levels of Bax, Bcl-2 and Cleaved Caspase-3 were measured by immunofluorescence (scale bar, 50 μm, n = 6). **D** Apoptosis of HK-2 cells was determined by TUNEL assays (Scale bar, 50 μm, n = 6). **H** The percentage of TUNEL-positive cells per field (× 400). HG: 30 mM d‐glucose, HG + C: HG + control pcDNA3.1(+), HG + NQO1 O/E: HG + NQO1 pcDNA3.1(+), HG + NQO1 O/E + EX527: HG + NQO1 pcDNA3.1(+) + EX527 (1 μM). Values are expressed as the mean ± SD. **P < 0.01 versus HG + C group; ^#^*P* < 0.05, ^##^*P* < 0.01 versus HG + NQO1 O/E group

In addition, we assessed the effect of EX527 on HG‐induced apoptosis in the HG + NQO1 O/E group. Compared with those in the HG group, the Bax/Bcl-2 protein ratio and Cleaved Caspase-3 level decreased in the HG + NQO1 O/E group. However, these changes were reversed by EX527 (Fig. 8A–C). Statistical analysis showed that the Bax/Bcl-2 ratio and Cleaved Caspase-3 level were upregulated by 3.34- and 1.75-fold, respectively, in the HG + NQO1 O/E + EX527 group compared with the HG + NQO1 O/E group (*P* < 0.05). Moreover, the expression of Bax, Bcl-2 and Cleaved Caspase-3 in HK-2 cells was analyzed by immunofluorescence, which confirmed the western blot results. EX527 treatment upregulated Bax and Cleaved Caspase-3 expression and downregulated Bcl-2 expression, as indicated by green granules, in HG-induced HK-2 cells transfected with the NQO1 pcDNA3.1(+) plasmid (Fig. 8D–G). Consistently, the TUNEL results revealed that the number of TUNEL-labeled cells in HG + NQO1O/E group was markedly less than that in the HG group; EX527 treatment decreased the quantity of TUNEL-labeled HK-2 cells in the HG + NQO1O/E group (Fig. 8D, H). These findings suggest that the antiapoptotic effect of NQO1 on HG-cultured HK-2 cells might be related to Sirt1.

### Protein expression of NQO1 in renal tissue samples from patients with DN

**Fig. 9:**
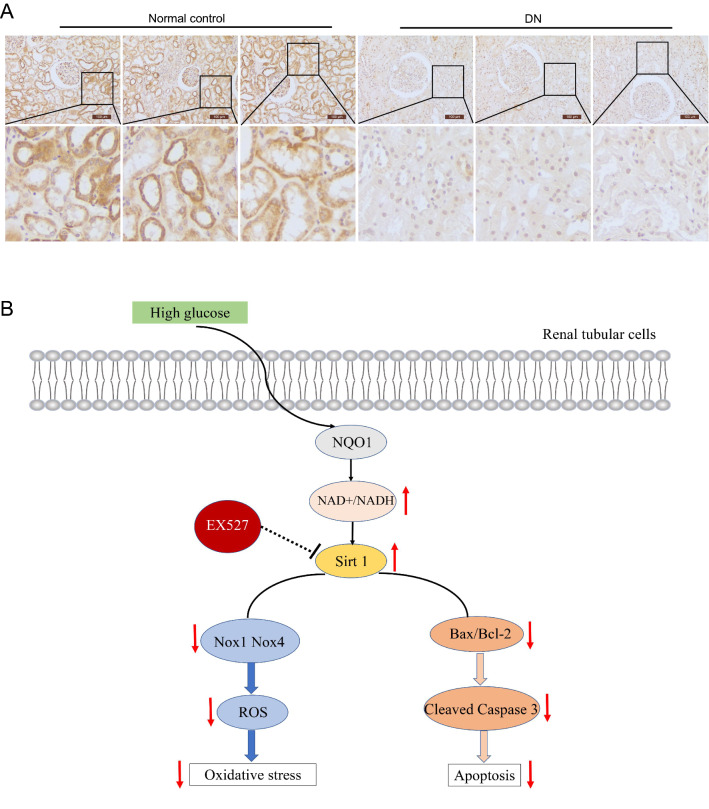
The expression of NQO1 in the kidneys of patients with DN. **A** Expression of NQO1 in the kidneys of patients with DN or in normal control kidneys was determined by immunohistochemical staining (Scale bar, 100 μm, n = 15). **B** Model showing the effect of NQO1-regulated NAD+/NADH on Sirt1-mediated oxidative stress and apoptosis in HG-induced renal tubular cells

Taken together, these findings revealed the antioxidative stress and antiapoptotic effects of NQO1 in vitro and in vivo. Further immunohistochemical staining was used to assess the expression level of NQO1 in renal biopsies from patients who were pathologically diagnosed with DN. The results showed that compared with that in the normal control group, the expression of NQO1 in the kidneys of patients with DN was significantly decreased (Fig. 9A). These observations were consistent with those in animal models and further supported the view that abnormal expression of NQO1 was related to the development of DN.

## Discussion

DN is a serious microvascular complication of diabetes and the main cause of chronic renal disease and ESRD [31, 32]. The pathogenesis of DN is a complex process involving multiple factors and many molecular mechanisms. Although glomerular injury is the focus of kidney injury, studies have shown that renal tubular damage plays an important role in the development of nephropathy (including DN). In our current study, we found that NQO1 was reduced in the kidneys of patients with type 2 diabetes mellitus with nephropathy and db/db mice, especially in renal tubules. Our results showed that NQO1 overexpression significantly attenuated renal oxidative stress and apoptosis and improved renal function in db/db mice. In addition, the in vitro results demonstrated that NQO1-mediated Sirt1 attenuated oxidative stress and apoptosis in HK-2 cells exposed to HG conditions.

NQO1 is an antioxidant enzyme downstream of the nuclear factor E2-related factor 2 (Nrf2)/antioxidant response element (ARE) signaling pathway. In our current study, we found that NQO1 was downregulated in diabetic renal tubular cells. Consistent with this finding, the expression of NQO1 in diabetic kidneys was reduced, and the upregulation of Nrf2/NQO1 could reduce diabetic kidney damage [33, 34]. Interestingly, a recent study reported that NQO1 expression was increased in the podocytes of diabetic mice stimulated with streptozotocin (STZ) for 8 weeks, while NQO1 deletion exacerbated diabetic kidney damage [35]. These studies indicated that the expression of NQO1 may fluctuate during different periods of DN. In summary, NQO1 may be a potential protective factor against DN.

Hyperglycemia promotes apoptosis in various types of cells in DN, including proximal tubular epithelial cells, but the mechanism is not fully clear [36]. Studies have shown that reducing renal tubulate epithelial cell apoptosis can improve related indicators of renal function, such as BUN and UACR, further preventing the occurrence and development of DN [37]. In the present study, the in vivo and in vitro results showed that NQO1 overexpression decreased Bax and Cleaved Caspase-3 expression and increased the expression of Bcl-2 in db/db mice and HK-2 cells cultured with HG. Consistent with this finding, the TUNEL results showed that NQO1 overexpression reduced renal cell apoptosis. Next, we evaluated the effect of NQO1 overexpression on renal function in db/db mice. The results showed that AAV-NQO1 treatment dramatically decreased UACR and BUN levels and improved renal function in db/db mice. Interestingly, similar results showed that in other renal disease models, such as ischemia–reperfusion injury (IRI) and cisplatin-induced acute renal injury, NQO1 blocks renal tubular cell apoptosis and improves renal function [38, 39]. These findings indicate that NQO1 overexpression can relieve renal damage in DN by inhibiting the apoptosis of renal tubular epithelial cells.

Oxidative stress is a key risk factor for many diseases, and antioxidant therapy is very important, including in DN [40–42]. Massive accumulation of ROS can promote renal tubular cell apoptosis and aggravate renal injury [43]. Nox isozymes have been proven to mediate the production of ROS via receptors and participate in the physiological processes of cell growth, differentiation, apoptosis and fibrosis [44]. NQO1 is a widely distributed flavoprotein that depends on FAD, and it functions as an antioxidant enzyme [45]. In our study, we found that Nox1, Nox4 and 8-OHdG expression was downregulated in db/db mice after treatment with AAV-NQO1. In addition, overexpressing NQO1 reduced ROS production and the protein levels of Nox1 and Nox4 in HG-exposed HK-2 cells. Similarly, previous studies have shown that NQO1 activation could inhibit ROS generation, which is related to Nox proteins in kidney injury induced by salt and cisplatin [22, 39]. These results indicate that NQO1 may have a renal protective effect against DN by inhibiting cell apoptosis through antioxidant effects.

NAD+ is the basic molecule of metabolism and redox signaling. The balance between NAD+ and NADH may be seriously disturbed because of diabetes mellitus and its complications. Many studies have shown that in various animal disease models, including cardiomyopathy [9], hearing impairment [46] and small intestinal injury [47], maintaining the balance of intracellular NAD+ is critical for cell survival. Activation of NQO1 can increase intracellular NAD+ , which may be a potential target for the treatment of various diseases. In the present study, we found that the NQO1 overexpression reversed the decrease in intracellular NAD+ levels caused by hyperglycemia.

Sirt1 is an NAD+ -dependent deacetylase that belongs to the Sirt family [48]. Previous studies have shown that overnutrition, such as diabetes, usually produces excessive NADH, and a decrease in NAD+ content often leads to a decrease in Sirt1 expression [49, 50]. Therefore, increasing the expression of Sirt1 in tissues extracted from diabetic animals has been considered a method to treat diabetes and its complications [51, 52]. In the current study, we demonstrated that NQO1 overexpression significantly increased the expression and activity of Sirt1 in vivo and in vitro. Our previous research indicated that SRT1720 can activate Sirt1 to reduce oxidative stress and renal fibrosis in DN [53]. Based on these research results, we hypothesize that NQO1 may activate Sirt1 to play a renal protective role by upregulating NAD+ levels. To verify the potential molecular mechanism of NQO1-mediated renal protection, we applied the Sirt1 inhibitor EX527 to HK-2 cells transfected with NQO1 pcDNA3.1(+) under HG conditions. The results showed that EX527 administration obviously enhanced ROS generation and the expression levels of Nox1 and Nox4 in HK-2 cells that were treated with NQO1 pcDNA3.1(+) under HG conditions. In addition, similar evidence has shown that dunnione ameliorates cisplatin-induced hearing loss by blocking oxidative stress, and this effect is mediated by the regulation of PARP-1 and Sirt1 through NQO1-mediated NADH oxidation [54]. Moreover, EX527 administration significantly upregulated the Bax/Bcl-2 ratio and Cleaved Caspase-3 expression in HK-2 cells transfected with NQO1 pcDNA3.1(+) under HG conditions. EX527 reversed the antiapoptotic and antioxidant effects of NQO1 overexpression on HK-2 cells cultured with HG, suggesting that NQO1 improves oxidative stress and apoptosis by regulating Sirt1 in DN.

In conclusion, our results showed that NQO1 overexpression improved oxidative stress and apoptosis in diabetic mouse kidneys and HG-cultured HK-2 cells. In addition, we found that the overexpression of NQO1 upregulated the NAD+/NADH ratio and Sirt1 expression, while inhibiting Sirt1 reversed the protective effect of NQO1 overexpression on HG-cultured HK-2 cells. These findings suggest that the protective effect of NQO1 may be achieved by activating sirt1 through the regulation of NAD+/NADH (Fig. 9B). Our findings reveal that NQO1 may be a potential target for the treatment of DN.

## Data Availability

The datasets used and/or analyzed during the current study are available from the corresponding authors on reasonable request.
